# Genome‐Wide Analyses for cognitive performances in Multi‐Ethnic Cohorts identifies novel rare locus *PCAT5*

**DOI:** 10.1002/alz70855_107089

**Published:** 2025-12-25

**Authors:** Neetesh Pandey, Sandra Barral, Richard Mayeux, Rafael Samper‐Ternent, Rebeca Wong, Giuseppe Tosto

**Affiliations:** ^1^ Columbia University, New York, NY, USA; ^2^ Gertrude H. Sergievsky Center, Columbia University Irving Medical Center, New York, NY, USA; ^3^ Departments of Neurology, Psychiatry, and Epidemiology, Gertrude H. Sergievsky Center, The Taub Institute for Research on Alzheimer's Disease and the Aging Brain, Vagelos College of Physicians and Surgeons, Columbia University, New York, NY, USA; ^4^ University of Texas Medical Branch, Galveston, TX, USA; ^5^ University of Texas, San Antonio, TX, USA; ^6^ Department of Neurology, College of Physicians and Surgeons, Columbia University, and the New York Presbyterian Hospital, New York, NY, USA

## Abstract

**Background:**

Employing a joint test of objectively‐measured endophenotypes vs. traditional binary outcomes (i.e., affected vs. non‐affected) can enhance power to discover novel genetic loci for Alzheimer's disease and related dementia (ADRD). We tested the association between rare variants and scores across multiple cognitive domains using data from the Mexican Health and Aging Study (MHAS) and conducted replication analyses in two multi‐ethnic cohorts: Washington Heights Inwood Aging Project (WHICAP) and Multi‐Ethnic Study of Atherosclerosis (MESA).

**Method:**

Genome‐wide gene‐based test employing rare variants from whole‐genome sequencing (WGS) was carried out using MultiSKAT package in 1,930 Mexicans. Replication used imputed genotype data from 909 WHICAP participants and 4,276 MESA participants. Variants were filtered on allele frequency (<1%) and, if imputed, >=80%. We adjusted for sex, age, education, and *APOE*.

**Result:**

In MHAS, we identified a genome‐wide significant locus, *PCAT5*, before and after *APOE* adjustment (*p* = 9.89E‐07; *p* = 9.74E‐07, respectively). We replicated the signal in both WHICAP (*p* = 0.018, *p* = 0.019, before/after *APOE* adjustment, respectively) and MESA (*p* = 4.07E‐10; *p* = 7.12E‐10 before/after APOE adjustment, respectively). Within the latter, when stratified by ethnicity, African Americans (*n* =  1,018) showed a nominal significant association before/after APOE adjustment (*p* = 0.0254; *p* = 0.0248, respectively).

**Conclusion:**

This study provides strong evidence for the association of rare variants within *PCAT5* and cognitive performances across different populations. Importantly, a rare variant in *PCAT5* was previously reported in a large meta‐analysis for ADRD of 65,602 Non‐Hispanic Whites (Naj, 2021). These findings suggest that *PCAT5* may play a role in ADRD pathology independently of *APOE*, warranting further biological investigations to understand the mechanisms underlining this gene's role.

